# A Real-World Evidence Study for Distribution of Traditional Chinese Medicine Syndrome and Its Elements on Respiratory Disease

**DOI:** 10.1155/2018/8305892

**Published:** 2018-12-12

**Authors:** Fei Xu, Wengqiang Cui, Qing Kong, Zihui Tang, Jingcheng Dong

**Affiliations:** ^1^Department of Integrative Medicine, Huashan Hospital, Fudan University, Shanghai, China; ^2^Institutes of Integrative Medicine, Fudan University, Shanghai, China; ^3^Department of Integrative Medicine and Neurobiology, State Key Laboratory of Medical Neurobiology, Institute of Acupuncture Research, School of Basic Medical Science, Fudan University, Shanghai, China

## Abstract

**Background:**

This study aimed to investigate the distribution and characteristics of traditional Chinese medicine (TCM) syndrome and its elements on respiratory diseases (RDs) based on real-world data (RWD).

**Methods:**

A real-world study was performed to explore the relationships among TCM syndrome and RDs based on electronic medical information. A total of 26,074 medical records with complete data were available for data analysis. Factor analyses were used to reduce dimensions of TCM syndrome elements and detect common factors. Additionally, cluster analyses were employed to assess combinations of TCM syndrome elements. Finally, association rule analyses were performed to investigate the structures of TCM syndrome elements to estimate the patterns of TCM syndrome.

**Results:**

A total of 27 TCM syndromes were extracted from RWD in this work. There were four TCM syndromes with >5.0% frequency based on the distribution frequency. The top five pathogenesis TCM syndrome elements were Tan, Huo, Feng, Qi_Xu, and Han. Factor analysis, cluster analysis, and association rule analysis demonstrated that Tan, Huo, Feng, Qi_Xu, Shen, and Fei were the core TCM syndrome elements.

**Conclusion:**

Four common Shi TCM syndromes on RDs were identified: Tan_Re_Yong_Fei, Tan_Zhuo_Zu_Fei, Feng_Re_Fan_Fei, and Feng_Han_Xi_Fei; two core common Xu TCM syndromes (Fei_Shen_Qi_Xu and Fei_Yin_Xu) and two core common Mix TCM syndromes (Fei_Pi_Qi_Xu-Tan_Shi_Yun_Fei and Fei_Shen_Qi_Xu-Tan_Yu_Zu_Fei) were also determined. The core TCM syndrome elements of Tan, Huo, Feng, Qi_Xu, Shen, and Fei were identified in this work.

## 1. Introduction

Traditional Chinese medicine (TCM) has progressively gained wider attention worldwide due to its specific theory and long historical clinical practice [1]. Throughout the world, TCM is increasingly being used for individuals by 10-20% annually [2]. TCM has obvious advantages of extending strength through the invigoration of the body and clearing the root of disease with fewer side effects and multi-target effects [3]. TCM has popularly been used to manage many diseases, such as inflammatory diseases, cancer, and respiratory disease [4–8].

In clinical practice, TCM practitioners form diagnoses and prepare prescriptions mainly on the basis of the pattern of the manifestation of symptoms that vary between each individual, which is known as TCM syndrome (also called “*ZHENG*”) [9]. TCM syndrome is a specific set of symptom or a pattern of symptoms presenting the body's internal and external condition at a certain stage [9]. Generally, it describes the patterns of bodily disharmony according to eight principles. In addition, it also differentiates syndromes according to another system: qi, blood, body-fluid differentiation, and zangfu (organ). TCM syndrome has successfully guided disease research and the prescribing of herbal formulas. Moreover, TCM syndrome, in conjunction with modern medicine diagnosis, is fundamental for diagnosis and treatments in China. Syndrome elements that are gained from four-step validation contribute to syndrome patterns. The syndrome elements are used for explaining TCM syndrome patterns and for reflecting the innate pathologic factors [10].

Respiratory diseases (RDs) are the major cause of mortality and morbidity worldwide, and they represent an enormous and increasing healthcare and economic burden, especially asthma, chronic obstructive pulmonary disease (COPD) and interstitial pulmonary disease (IPD) [11]. Modern medicine therapies could not reverse all the symptoms, and the current available drugs may induce prominent side effects such as osteoporosis [12]. Generally, in China, TCM treatment for RDs has a long history, and numerous basic and clinical studies have assured that TCM has curative effects [13, 14].

Evidence from a real-world study, outside of randomized clinical trials (RCT), is considered as a way to tailor medical decision-making more closely to the characteristics of individuals for making clinical practice more personalized and effective [15]. Real-world evidence (RWE) is derived from data associated with outcomes from the care of heterogeneous patients as experienced in real-world practice settings. Fortunately, big data methods could effectively manage and treat massive-scale, multiple-source, and heterogeneous real-world data (RWD) [15]. Moreover, data mining or machine learning algorithms, such as factor analysis, cluster analysis, and association rule analysis, could analyze and model the data for RWE. RWE studies will never replace the more robust RCT; however, the emerging trend is to incorporate evidence to be of benefit to medical decision-making. Importantly, it is crucial to perform real-world studies on TCM syndrome to accumulate RWE based on RWD by using big data with data mining or machine learn algorithms.

Currently, studies on TCM syndrome differentiation have been widely developed. However, those pertaining to RDs have not been explored based on RWD through the use of big data methods and data mining algorithms. This study aimed to investigate the distribution and characteristics of TCM syndrome and its elements relating to whole RDs based on real-world datasets.

## 2. Methods

### 2.1. Study Design and Participants

A real-world study was performed to explore the relationships among TCM syndrome and internal diseases based on electronic medical information including electronic medical records (EMR), hospital information system (HIS), laboratory information system (LIS), and picture archiving and communication system (PACS). In this work, between 2012 and 2016, all of 30,254 records were collected from respiratory disease wards in five hospitals: Department of Integrative Medicine of Hushan Hospital of Fudan University, Puer Hospital of Chinese Medicine, Danyang Hospital of Chinese Medicine, Changde Hospital of Hunan University of Chinese Medicine, and Ruikang Hospital of Guangxi University of Chinese Medicine. Ethics approval of the present study was given by the Ethical Committee of the Huashan Hospital. 

Inclusion criteria were as follows: diagnoses satisfying the diagnostic criteria of modern medicine for respiratory diseases with detailed medical records, a first final diagnosis of a respiratory disease and subjects being over the age of 18. Exclusion criteria were as follows: unclear diagnoses or diagnoses not satisfying the diagnostic criteria of modern medicine for respiratory diseases, subjects not satisfying the age criteria, and unclear or incomplete medical records.

### 2.2. Data Collection and Preparation

The content of medical records for patients was based on EMR and standard Chinese guidelines. The content mainly included general information, complaints, medical histories, modern medicine diagnoses, and TCM diagnosis. Data regarding medical records for patients were transferred from EMR systems of five hospitals and loaded to medical big data platforms of institutes of biomedical informatics and biostatistics and institutes of integrative medicine of Fudan University.

In this work, RDs included chronic obstructive pulmonary disease (COPD), lung infection, chronic bronchitis, lung cancer, acute bronchitis, bronchial asthma, bronchiectasis, acute upper respiratory tract infection, pulmonary tuberculosis, pleural fluid, interstitial lung disease, pleurisy, pulmonary heart disease, pneumoconiosis, pneumothorax, lung abscess, pulmonary encephalopathy and pulmonary embolism. Diagnosis criteria of these RDs were based on the Chinese Society of Respiratory Diseases, the Chinese Medicine Association and its guidelines, and the book* Harrison's Principles of Internal Medicine* [16, 17].

**Figure 1:**
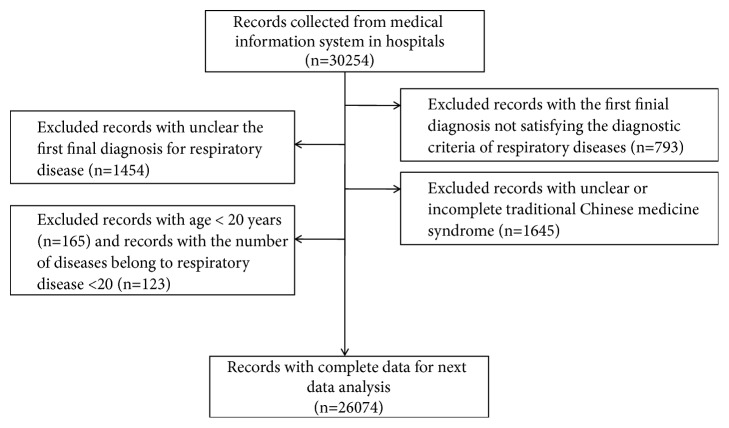
Flow chat of study data collection and the number of individuals for data analysis.

The general information (age, gender, entrance time of hospitalization, duration of hospitalization, and clinical outcomes), TCM syndrome, TCM diagnosis, the first final modern medicine diagnosis, and additional modern medicine diagnoses were extracted by using Python 3.5 programs. Standard common data models (CDM) for RDs of integrative medicine including standard TCM syndrome were created to uniform standard code for data analysis [9]. An integrative medicine data warehouse for RD research was created based on standard CDM and big data platform. A total of 26,074medical records with complete data consisting of general information, TCM syndrome, and TCM and final modern medicine diagnoses were available for future data analysis (Figure 1).

### 2.3. Data Analysis

In this work, these main data analyses are conducted to assess the rule of distribution of TCM syndrome and its elements, including the following: (1) frequency distribution of TCM syndrome; (2) frequency distribution of TCM syndrome elements; (3) the combination of TCM syndrome elements based on data mining algorithms; and (4) consistent analysis of TCM syndrome and a combinations of its elements. Differences in variables among subjects grouped by gender were determined by one-way analysis of variance. Among the groups, differences in properties were detected by *χ*^2^ analysis. Tests were two-sided, and a p-value of < 0.05 was considered significant. Frequency analyses were employed to explore the proportion of RDs and proportion of TCM syndrome for respiratory systems. Elements of TCM syndrome were generated according to standard TCM syndrome and its element guidelines [10]. Moreover, frequency analyses were performed to assess the proportion of TCM syndrome elements. Results were analyzed using the Statistical Package for Social Sciences for Windows, version 16.0 (SPSS, Chicago, IL, USA).

Factor analyses were used to reduce dimensions of TCM syndrome elements and detect the structure among these TCM syndrome elements. The Kaiser-Meyer-Olkin (KMO) test and Bartlett's test of sphericity were used to evaluate suitability of collected TCM syndrome elements for factor analysis [18]. Principal component analyses were applied to extract common factors [18]. Varimax rotation was used to allow the factor load absolute value of the new common factor [18]. In this work, factor load absolute value was larger or equal to 0.20. Cluster analyses were employed to classify TCM syndrome elements. Hierarchical cluster analyses were conducted using Ward's method to generate a dendrogram for estimation of the similar clusters. Cluster boundaries were defined by large distances between successive fusion levels [19].

Additionally, considering the complex network structure for TCM syndrome elements, association rule analyses were performed to investigate the structures of TCM syndrome elements to estimate the distribution of TCM syndrome. A set of frequent rules is generated, and the strength of the rules that was obtained from the first stage is then evaluated [20]. The Apriori algorithms were used to evaluate the pattern of association within TCM syndrome elements. Three parameters of support, confidence and lift were used to assess the strength of rules [20]. Let X be an item set, X=>Y an association rule, and T a set of transactions of a given dataset. Support is an indication of how frequently the item set appears in the dataset, defined as the probability of transactions in T containing X and Y. Confidence is an indication of how often the rule has been found to be true, defined as the conditional probability of having Y given X. Lift is the ratio of the observed support to be expected if X and Y were independent. Lift values of <1, 1, and >1 signify the negative, independent, and positive associations between X and Y, respectively. In this work, rules having a support% value of >10 and confidence% value of >80 were reported. Data mining was performed using the SPSS Modeller (version 18.0, Chicago, IL, USA) and packages in Python 3.5.

## 3. Results

### 3.1. Characteristics of Individuals

The baseline characteristics of the 26,074individuals are listed in Table 1. In the entire dataset, the proportion of males (n=15350) was 58.87%, and the mean age was 65.70 years. The average duration of hospitalization was 11.86 days. Males had more days of hospitalization than females (12.79 vs. 10.53, P<0.001). The major ethnicity for the dataset was Chinese Han (94.76%). The rate of improvement and being cured for patients was 95.16%.

### 3.2. Frequency Analysis of Respiratory Diseases

Distribution of RD in the total sample was listed in Table 2. The main 16 diseases were analyzed in this work. COPD and lung infection were proportionally the two highest diseases in hospitals (32.05% for COPD and 27.81% for lung infection). The proportion of chronic bronchitis and lung cancer was 8.46% and 7.33%, respectively.

### 3.3. Frequency Analysis of TCM Syndrome

Distribution of TCM syndrome for RD was listed in Table 3. A total of 27 TCM syndromes were extracted in this work. The top five proportions of Shi TCM syndrome were Tan_Re_Yong_Fei, Tan_Zhuo_Zu_Fei, Feng_Re_Fan_Fei, Feng_Han_Xi_Fei, and Shui_Ling_Xin_Fei (27.61%, 25.60%, 10.49%, 6.81%, and 3.45% for the five TCM syndromes, respectively). The top four proportions of Xu TCM syndrome were Fei_Shen_Qi_Xu, Fei_Yin_Xu, Fei_Shen_Ying_Xu, and Fei_Qi_Xu (5.80%, 5.60%, 1.22%, and 0.69 for the four TCM syndromes, respectively). Additionally, the top three proportions of mixed TCM syndrome were Fei_Pi_Qi_Xu-Tan_Shi_Yun_Fei, Fei_Shen_Qi_Xu-Tan_Yu_Zu_Fei, and Fei_Pi_Qi_Xu-Tan_Yu_Zu_Fei (1.37%, 1.37%, and 1.17% for the three TCM syndromes, respectively).

### 3.4. Frequency of TCM Syndrome Elements

A total of 20 elements generated from the 27 TCM syndromes were listed in Table 4. The top five pathogenesis TCM syndrome elements were Tan, Huo, Feng, Qi_Xu, and Han (22.09%, 14.75%, 6.39%, 5.09%, and 3.09% for the five TCM syndrome elements, respectively), while the top location TCM syndrome elements were Fei, Shen, Pi, and Biao (35.08%, 3.45%, 1.09%, and 0.62% for the four TCM syndrome elements, respectively).

### 3.5. Factor Analysis of TCM Syndrome Elements

In entire sample, the KMO value of the partial correlation of variables was 0.613, and the approximate chi-square value of Bartlett's test of sphericity was 712.34 (P<0.001), indicating strong correlation between variables and indicating that the variables could be applied to factor analysis. Similar results were reported in Shi, Xu, and Mix groups (KMO>0.50 and P for Bartlett's test <0.001 for all).

**Figure 2:**
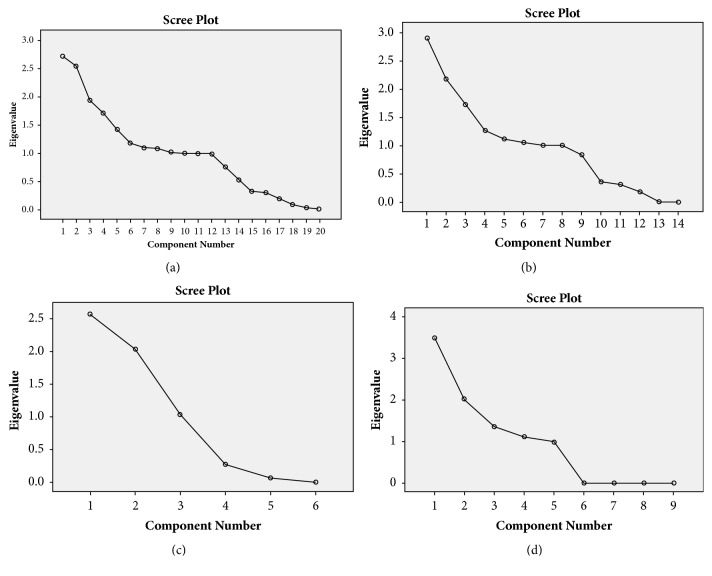
Scree plot of characteristic root value of common factors. (a) Scree plot of characteristic root value of common factors for entire dataset; (b) Scree plot of characteristic root value of common factors for Shi TCM syndrome group; (c) Scree plot of characteristic root value of common factors for Xu TCM syndrome group; (d) Scree plot of characteristic root value of common factors for Mix TCM syndrome group.

In the entire sample, principal component analysis showed that characteristic root values of the first 10 common factors were greater than 1.0, and their cumulative variance contribution rates reached 78.91. A Scree Plot displayed relevance of common factors and characteristic root values (Figure 2(a)), indicating that the scatter location of the first 10 common factors was steep and that characteristic root values of the rest of the common factors were small. Varimax rotation was used for factor rotation and transformation, and absolute factor load values that were larger or equal to 0.20 were listed in Table 5. Similarly, eight common factors, three common factors, and four common factors were extracted from Shi, Xu, and Mix TCM syndrome groups, respectively (Figures 2(b)–2(d)). Furthermore, results of the factor load matrix after rotation transformation were listed in Table 5.

Generally, the Shi TCM syndrome patterns to include Tan_Re_Yong_Fei, Tan_Zhuo_Zu_Fei, Feng_Re_Fan_Fei, and Feng_Han_Xi_Fei were established, while the Xu TCM syndrome patterns to include Fei_Shen_Qi_Xu, Fei_Yin_Xu, and Shen_Qi_Xu were established. The Mix TCM syndrome of Fei_Shen_Qi_Xu-Tan_Yu_Zu_Fei was established.

### 3.6. Cluster Analysis for TCM Elements

**Figure 3:**
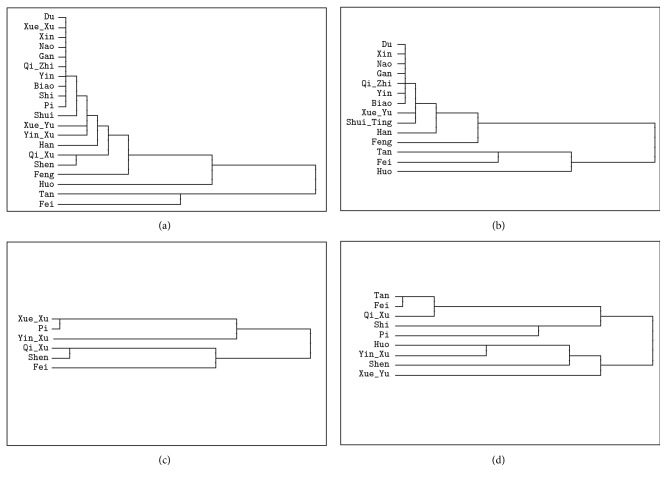
Cluster analysis for traditional Chinese medicine (TCM) syndrome element on respiratory disease. (a) Results of cluster analysis for TCM syndrome element in entire dataset; (b) results of cluster analysis for TCM syndrome element in Shi TCM syndrome group; (c) results of cluster analysis for TCM syndrome element in Xu TCM syndrome group; (d) results of cluster analysis for TCM syndrome element in Mix TCM syndrome group.

In the entire sample, hierarchical cluster analysis signified that these TCM syndrome elements were significantly different among three clusters (Figure 3). Cluster 1 comprised Huo, Tan and Fei, and Cluster 2 comprised Shen, Qi_Xu, Han, Yin_Xu, and Xue_Xu, while Cluster 3 comprised the rest of the TCM syndrome elements. In the Shi TCM syndrome group, three clusters were identified, suggesting that Cluster 1 included Tan, Fei, and Huo, Cluster 2 included Feng, Han, and Shui_Ting, and the rest of the TCM syndrome elements belonged to Cluster 3. In the Xu TCM syndrome group, two clusters were generated, indicating that Cluster 1 consisted of Fei, Shen, Qi_Xu, and Yin_Xu and that Cluster 2 consisted of Pi and Xue_Xu. In the Mix TCM syndrome groups, Cluster 1 included Huo, Yin_Xu, Shen, and Xue_Yu, while the rest of the TCM syndrome belonged to Cluster 2.

### 3.7. Association Rule Analysis for TCM Elements

In the entire dataset, four rules satisfying rule algorithms were listed in Table 6. The strongest support% parameter (60.907) was between Tan and Fei. Three rules concerning Huo => Fei, Huo => Fei, and Huo and Tan => Fei had the strongest confidence% value (100). The association rule analysis informed the combinations among Tan, Huo, Feng, Qi_Xu, and Fei (Table 6). In the Shi TCM syndrome group, five rules had been identified, suggesting the combinations among Tan, Huo, Feng and Fei. Seven rules were reported in the Xu TCM syndrome groups, indicating the combinations among Qi_Xu, Yin_Xu, Shen, and Fei. In the Mix TCM syndrome group, the combinations among Qi_Xu, Tan, and Fei were reported. In general, association rule analysis demonstrated that Tan, Huo, Feng, Qi_Xu, Shen, and Fei were the core TCM syndrome elements.

## 4. Discussion

We conducted a real-world study to investigate the distribution and characteristics of TCM syndrome and its elements on RDs based on RWD. RCTs are the gold standard in the generation of medical evidence; however, they are being challenged by enrollment criteria, timelines and atypical comparators [15]. RWE are seen as complementary evidence generated from RCTs. RWE studies are increasingly becoming the normal practice in ensuring its significance in clinical practice. More importantly, big data methods have the advantage of managing and collecting massive-scale, real-world medical data [15]. Furthermore, the methods could effectively transform, extract and treat the multiple-sources and heterogeneous RWD to a standard structured dataset. In this work, standard CDM data was used for data analysis. Factor analysis, cluster analysis and association rule analysis were used to explore common factors and evaluate the combined pattern for TCM syndrome with its elements. To our best knowledge, this is the first real-world study in China to investigate distribution and characteristics of TCM syndrome and its elements based on RWD by using big data methods and data mining or machine learning algorithms. In addition, this study also provided valuable experience in real-world study for clinical research on TCM.

In this study, the two most frequent RDs were COPD (32.05%) and lung infection (27.81%).Our findings indicated that four common syndromes in decreasing order of frequency were Tan_Re_Yong_Fei, Tan_Zhuo_Zu_Fei, Feng_Re_Fan_Fei, and Feng_Han_Xi_Fei. Frequency analyses showed the four Shi TCM syndromes with >5.0% frequency. In the Shi TCM syndrome group, factor analyses also indicated the four common syndromes for RDS. Cluster analyses and association rule analyses presented the combinations among Tan, Huo, Feng, Han, and Fei, suggesting that the four TCM syndromes were the common ones for RDs. Similarly, in the Xu TCM syndrome group, Fei_Shen_Qi_Xu and Fei_Yin_Xu were two core common TCM syndromes, and among the Mix TCM syndromes, Fei_Pi_Qi_Xu-Tan_Shi_Yun_Fei was the most common. Additionally, frequency analyses, factor analyses, cluster analyses, and association rule analyses for TCM syndrome elements demonstrated that the predominant elements in the pathogenesis of RD were Tan, Huo, Feng, and Qi_Xu. The main disease location is Fei and Shen.

Currently, a standard TCM syndrome differentiation of some RDs has not yet been established, such as COPD. In general, the standard TCM syndrome differentiation was just primarily established from the literature analysis and expert counseling, and there is limited evidence for clinical application. In this study, data collected from 26,074medical case records reflected the clinical common syndromes of RDs, which provided powerful evidence for a standard TCM syndrome differentiation. In TCM, syndrome pattern is the principle for treatment, and more attention should then be paid to the accuracy of syndrome classification. In this study, the abovementioned eight syndrome patterns may provide a guideline for differentiation and treatment for RDs.

Syndrome elements are the smallest unit of syndrome patterns explaining complexity and flexibility of TCM syndrome differentiation and reflecting the innate pathologic factors [10]. The real-world study demonstrated that lungs were more easily attacked by Tan, Huo, and Feng. Pathogenic Feng (wind) is the leading pathological cause of all diseases. When the defense system is weak and the protective Qi loses its ability to protect the body from foreign pathogens, which are often attached with other morbidity factors, such foreign pathogens invade the human body [21]. Phlegm syndrome appears in almost all RDs. For instance, frequency of phlegm in COPD is almost 60% [22]. The pathogenic factors may disrupt the function of lungs, block Qi, and blood circulation, influence the function of organs, and cause numerous negative changes in the body. Thus, phlegm is the pathological product of the disturbance of body fluid in the transportation and stagnation of body fluids [23]. Moreover, phlegm syndrome is almost always accompanied by other syndromes, such as phlegm-damp, phlegm-heat and phlegm-stasis syndromes [12, 24]. Consistent with the previous findings, these syndrome elements, Tan (damp), Huo (heat), Feng (stasis.) and Shui_Ting, appeared frequently in RDs in the present study.

Although Shi syndromes had the highest distribution frequency, Xu syndromes appear throughout the whole course of disease. Deficiency in origin mainly included lung and Qi deficiency; as the disease continued to advance, Yin and Pi deficiency appeared. In a long course, Shen was involved and insufficiently presented. If it brought about Yin deficiency of Shen, it was not nourishing Gan wood and finally led to Gan and Shen deficiency and prosperity of fire [25]. When the syndrome was accurately differentiated and distinguished, the primary and secondary symptoms were taken into account. For example, a clinical investigation of 2,500 adult asthma cases identified the common ZHENG with primary and secondary symptoms [24]. Interestingly, these phenomena were in accordance with the analysis results of syndromes and elements frequency distribution.

In spite of the profound significance of this study, there are some limitations of this study. Firstly, the present study was based on real-world study design, so selection bias could not be avoided. The multiple-center and massive-scale cases have been collected that can reduce the bias. Secondly, the multiple-sources and heterogeneous medical information could make data analysis difficultly. Fortunately, big data and data mining or machine learning algorithms could effectively transform, extract and treat them to generate standard CDM data. Thirdly, this work just explored the distribution of TCM syndrome and its elements on the whole level for RDs. In near future, those works for each disease concerning respiratory system should be performed separately. All of the limitations underscored the importance of this study, and the results of this study made a significant contribution to the standardization of RD syndromes and treatment.

## 5. Conclusion

In summary, the distribution of TCM syndromes and its elements are identified through a real-world study. The overall 20 syndrome elements of whole RDs are combined into eight main syndromes, including Shi, Xu. and Mix syndromes. The significant five syndrome elements, Tan, Huo, Feng, Qi_Xu Shen, and Fei, are the basis of syndrome differentiation for RDs and created a bridge to the standardization of RD syndromes. Additionally, this study demonstrated the close correlation of some elements.

## Figures and Tables

**Table 1:** Baseline characteristics of individuals with respiratory diseases.

Variable	Male	Female	Total	P value^∗∗∗^
N	15350	10724	26074	-
Age	66.87±15.80	64.02±16.83	65.7±16.29	<0.001
Days of hospitalization	12.79±9.69	10.53±8.92	11.86±8.51	<0.001
Married, Yes (%)	14297(93.14)	9752(90.94)	24038(92.19)	<0.001
Ethnic, Han (%)	14516(94.57)	10189(95.01)	24707(94.76)	0.249
Entrance^∗^, (%)	14835(96.64)	10444(97.39)	25282(96.96)	0.012
Outcome^∗∗^, (%)	14501(94.47)	10292(95.97)	24812(95.16)	<0.001

*Note.* ^∗^entrance from outpatient clinic, ^∗∗^outcome concerning improved or cured individuals, and ^∗∗∗^difference analyses for variables between male and female.

**Table 2:** Distribution of respiratory disease in total sample.

ID	Disease	Frequency	Percent (%)
1	Chronic obstructive pulmonary disease	8358	32.05
2	Lung infection	7250	27.81
3	Chronic bronchitis	2207	8.46
4	Lung cancer	1911	7.33
5	Acute bronchitis	1395	5.35
6	Bronchial asthma	1346	5.16
7	Bronchiectasis	1113	4.27
8	Acute upper respiratory tract infection	759	2.91
9	Pulmonary tuberculosis	577	2.21
10	Pleural fluid	304	1.17
11	Interstitial lung disease	282	1.08
12	Pleurisy	167	0.64
13	Pulmonary heart disease	159	0.61
14	Pneumoconiosis	95	0.36
15	Pneumothorax	52	0.20
16	Lung abscess	36	0.14
17	Pulmonary encephalopathy	32	0.12
18	Pulmonary embolism	31	0.12
	Total	26074	100.00

**Table 3:** Distribution of traditional Chinese medicine syndrome pattern for respiratory disease in total sample.

ID	TCM syndrome type	TCM syndrome pattern	Frequency	Percent (%)
1	Shi	Tan_Re_Yong_Fei	7198	27.61
2		Tan_Zhuo_Zu_Fei	6674	25.60
3		Feng_Re_Fan_Fei	2734	10.49
4		Feng_Han_Xi_Fei	1776	6.81
5		Shui_Ling_Xin_Fei	899	3.45
6		Tan_Yu_Zu_Fei	449	1.72
7		Wai_Han_Nei_Yin	274	1.05
8		Qi_Zhi_Xue_Yu	253	0.97
9		Gan_Huo_Fan_Fei	246	0.94
10		Biao_Han_Fei_Re	170	0.65
11		Tan_Meng_Shen_Qiao	81	0.31
12		Feng_Tan_Zu_Fei	80	0.31
13		Qi_Zhi_Xin_Xiong	78	0.30
14		Re_Du_Yong_Fei	54	0.21

15	Xu	Fei_Shen_Qi_Xu	1513	5.80
16		Fei_Yin_Xu	1459	5.60
17		Fei_Shen_Qi_Yin_Liang_Xu	317	1.22
18		Fei_Qi_Xu	181	0.69
19		Qi_Xue_Kui_Xu	64	0.25
20		Fei_Pi_Qi_Xu	49	0.19

21	Mix	Fei_Pi_Qi_Xu_Tan_Shi_Yun_Fei	358	1.37
22		Fei_Shen_Qi_Xu_Tan_Yu_Zu_Fei	356	1.37
23		Fei_Pi_Qi_Xu_Tan_Yu_Zu_Fei	304	1.17
24		Fei_Shen_Qi_Xu_Tan_Shi_Yun_Fei	172	0.66
25		Qi_Yin_Liang_Yu_Xue_Nei_Zu	149	0.57
26		Fei_Shen_Qi_Xu_Tan_Re_Yong_Fei	117	0.45
27		Fei_Pi_Qi_Xu_Tan_Re_Yong_Fei	69	0.26

	Total		26074	100.00

*Note.* TCM: traditional Chinese medicine.

**Table 4:** Distribution of traditional Chinese medicine syndrome element for respiratory disease in total sample.

ID	TCM syndrome element type	TCM syndrome element	Frequency	Percent (%)
1	Pathogenesis	Tan	15859	22.09
2		Huo	10588	14.75
3		Feng	4590	6.39
4		Qi_Xu	3650	5.09
5		Han	2220	3.09
6		Yin_Xu	1925	2.68
7		Xue_Yu	1512	2.11
8		Shui_Ting	899	1.25
9		Shi	530	0.74
10		Qi_Zhi	332	0.46
11		Yin	274	0.38
12		Xue_Xu	64	0.09
13		Du	54	0.08

14	Location	Fei	25177	35.08
15		Shen	2475	3.45
16		Pi	781	1.09
17		Biao	444	0.62
18		Gan	246	0.34
19		Nao	81	0.11
20		Xin	78	0.11

	Total		71779	100.00

*Note.* TCM: traditional Chinese medicine.

**Table 5:** Common factors and their corresponding traditional Chinese medicine syndrome elements of interpretation of cause, and location of disease.

Group	Common factor	TCM syndrome element varibale of interpretation of cause	Location of disease
Entire	F1	Yin(0.935); Biao(0.927); Han(0.439)	Fei(-0.535);
	F2	Qi_Zhi(0.913); Xin(0.724); Xue_Yu(0.429)	Fei(-0.622);
	F3	Qi_Xu(0.880); Tan(-0.280); Huo(-0.257)	Shen(0.946);
	F4	Feng(0.918); Tan(-0.734); Han(0.714)	Fei(-0.764);
	F5	Qi_Xu(0.389); Shi(0.823)	Pi(0.901);
	F6	Xue_Yu(0.258); Tan(-0.365); Yin_Xu(0.856); Huo(-0.496)	
	F7	Xue_Xu(0.711);	Nao(0.677)
	F8	Xue_Yu(0.334); Tan(0.26); Huo(0.295); Shui_Ting(-0.89)	
	F9	Huo(0.385)	Gan(0.886);
	F10	Du(0.958)	

Shi	F1	Yin(0.941); Biao(0.925); Han(0.423)	Fei(-0.617)
	F2	Feng(0.938); Tan(-0.882); Han(0.699)	
	F3	Xue_Yu(0.927); Qi_Zhi(0.675); Huo(-0.331)	
	F4	Qi_Zhi(0.666); Xin(0.919)	Fei(-0.469)
	F5	Tan(-0.263); Shui_Ting(0.925); Huo(-0.54)	
	F6	Tan(0.993)	Fei(-0.369)
	F7	Tan(-0.238); Han(-0.238); Huo(0.463);	Gan(0.884)
	F8	Huo(0.202); Du(0.974)	

Xu	F1	Qi_Xu(0.967);Yin_Xu(-0.894)	Shen(0.91)
	F2	Qi_Xu(-0.998); Xue_Xu(0.232)	Fei(0.998); Shen(0.207)
	F3	Yin_Xu(-0.206)	Pi(0.994); Shen(-0.212)

Mix	F1	Yin_Xu(-0.992); Tan(0.992); Shi(0.296); Qi_Xu(-0.235);	Fei(0.992); Shen(0.297);
	F2	Shi(0.943); Xue_Yu(-0.895);	Pi(0.204);
	F3	Qi_Xu(0.217);	Shen(-0.937); Pi(0.928);
	F4	Shi(-0.269); Xue_Yu(-0.395); Huo(0.995);	

*Note.* Values in parentheses are results of factor load matrix after rotation transformation, absolute factor load values that were larger or equal to 0.20.

**Table 6:** Association rule analysis for traditional Chinese medicine syndrome element on respiratory disease.

Group	Consequent	Antecedent	Support %	Confidence %	Lift
Entire	Fei	Tan	60.91	99.49	1.04
	Fei	Huo	40.66	100.00	1.03
	Fei	Huo and Tan	28.36	100.00	1.03
	Fei	Feng	17.63	100.00	1.03

Shi	Fei	Tan	69.07	99.44	1.03
	Fei	Huo	49.61	100.00	1.03
	Fei	Huo and Tan	34.33	100.00	1.03
	Fei	Feng	21.89	100.00	1.03
	Fei	Feng and Huo	13.04	100.00	1.03

Xu	Shen	Qi_Xu	59.28	86.16	1.68
	Shen	Qi_Xu and Fei	57.49	88.84	1.74
	Qi_Xu	Shen	51.08	100.00	1.69
	Fei	Shen	51.08	100.00	1.02
	Fei	Shen and Qi_Xu	51.08	100.00	1.02
	Qi_Xu	Shen and Fei	51.08	100.00	1.69
	Fei	Yin_Xu	49.57	100.00	1.02

Mix	Fei	Tan	90.23	100.00	1.11
	Fei	Tan and Qi_Xu	90.23	100.00	1.11
	Qi_Xu	Tan and Fei	90.23	100.00	1.11
	Qi_Xu	Fei	90.23	100.00	1.11
	Tan	Fei	90.23	100.00	1.11
	Tan	Fei and Qi_Xu	90.23	100.00	1.11

*Note.* Support %> 10% and confidence% > 80%.

## Data Availability

The data used to support the findings of this study are available from the corresponding author upon request.

## Additional Points

Items interpretation is as follows: Fei: the lung; Pi: the pleen; Shen: the kidney; Xin: the heart; Nao: the brain; Shi: the excess; Xu: the deficiency; Huo: the fire; Tan: the phlegm; Feng: the wind.
